# Surface pretreatment for prolonged survival of cemented tibial prosthesis components: full- vs. surface-cementation technique

**DOI:** 10.1186/1475-925X-4-61

**Published:** 2005-10-31

**Authors:** Rudolf Marx, Mutaz Qunaibi, Dieter Christian Wirtz, Fritz Uwe Niethard, Thorsten Mumme

**Affiliations:** 1 Department of Prosthetic Dentistry, Section of Dental Materials, University Hospital of the University of Technology, Aachen, Germany; 2 Department of Orthopedic Surgery, University Hospital of the University of Technology, Aachen, Germany

## Abstract

**Background:**

One of few persisting problems of cemented total knee arthroplasty (TKA) is aseptic loosening of tibial component due to degradation of the interface between bone cement and metallic tibial shaft component, particularly for surface cemented tibial components. Surface cementation technique has important clinical meaning in case of revision and for avoidance of stress shielding. Degradation of the interface between bone cement and bone may be a secondary effect due to excessive crack formation in bone cement starting at the opposite metallic surface.

**Methods:**

This study was done to prove crack formation in the bone cement near the metallic surface when this is not coated. We propose a newly developed coating process by PVD layering with SiO_x _to avoid that crack formation in the bone cement. A biomechanical model for vibration fatigue test was done to simulate the physiological and biomechanical conditions of the human knee joint and to prove excessive crack formation.

**Results:**

It was found that coated tibial components showed a highly significant reduction of cement cracking near the interface metal/bone cement (p < 0.01) and a significant reduction of gap formation in the interface metal-to-bone cement (p < 0.05).

**Conclusion:**

Coating dramatically reduces hydrolytic- and stress-related crack formation at the prosthesis interface metal/bone cement. This leads to a more homogenous load transfer into the cement mantle which should reduce the frequency of loosening in the interfaces metal/bone cement/bone. With surface coating of the tibial component it should become possible that surface cemented TKAs reveal similar loosening rates as TKAs both surface and stem cemented. This would be an important clinical advantage since it is believed that surface cementing reduces metaphyseal bone loss in case of revision and stress shielding for better bone health.

## Background

One of the few still persistent problems of cemented TKA is aseptic loosening of the cemented prosthesis components due to hydrolytic degradation of the interface between bone cement and metal and/or bone, respectively. Thanks to improved surgical techniques, continued development of arthroplasty components and innovative fixation methods for the cemented components, long-time survival rates of at least 90% are reported for 10 to 15 years of follow-up [1,2]. Therefore, total knee arthroplasty is a highly successful procedure, with the percentage of patients requiring revision relatively small [4-7]. However, when considering the large number of these procedures performed annually, a small percentage of failures making revision necessary constitutes a significant number of patients. Annually, 22,000 TKAs are revised in the USA [1] and 35,000 TKAs are revised worldwide [2]. Failure of total knee arthroplasty is devastating to the patient and frustrating for the surgeon. The cost associated with revision surgery is substantial.

The leading causes for failure of TKAs are (only rates above 15 % are quoted) polyethylene wear (25 %), *aseptic loosening *(24.1 %), instability (21.2 %) and infection (17.5%) [3]. That rate for aseptic loosening (24.1 %) comprises loosening of cemented and uncemented TKAs, the figure for loosening of *un*cemented TKAs is about 21 % and the figure for surface cementing technique (the undersurface of the tibial base plate is cemented but the stem (keel) is not cemented) is about 10.5 % after an average interval of four years [1].

Under long-term clinical aspects surface cementing technique with press-fit seems to be particularly adequate and the restricted cemented area should be an important advantage. Although the cement application area is limited to the surface sufficient stability is believed to be achievable. This question, however, is controversially disputed in literature, since the loosening rate associated with surface cemented components is with 10.5 % [1] significantly larger than the overall loosening rate for cemented tibial components (3 %; [3]).

On revision fully cemented stems are difficult to remove and revision is accompanied by increased metaphyseal bone loss. Moreover, fully cemented stems promote stress shielding because the tight contact of bone cement and bone in the proximal region of the tibia means additional stiffness which is not physiological [8]. Stress shielding can cause an osteopenia and osteolysis type of bone loss and disuse osteoporosis [9,10]. Therefore, cement application restricted to the surface area may become the technique of fixation of choice in total knee arthroplasty [8] provided the increased rates of aseptic loosening corresponding to this technique can be reduced. This is our goal.

The question of cemented vs. cement-free components is here not addressed. Particularly regarding the *tibia *plateau cement-free tibial components have poor primary stability (microscopic movements in the range of 30 – 100 μm may occur) and this hinders bone from growing in. Thus the cemented fixation is considered to be the golden standard for tibial components provided a technology is available which improves the stability of only surface cemented tibia components without partially or completely filling up the space between stem and bone with bone cement. Instead press-fit is applied.

**Figure 3:**
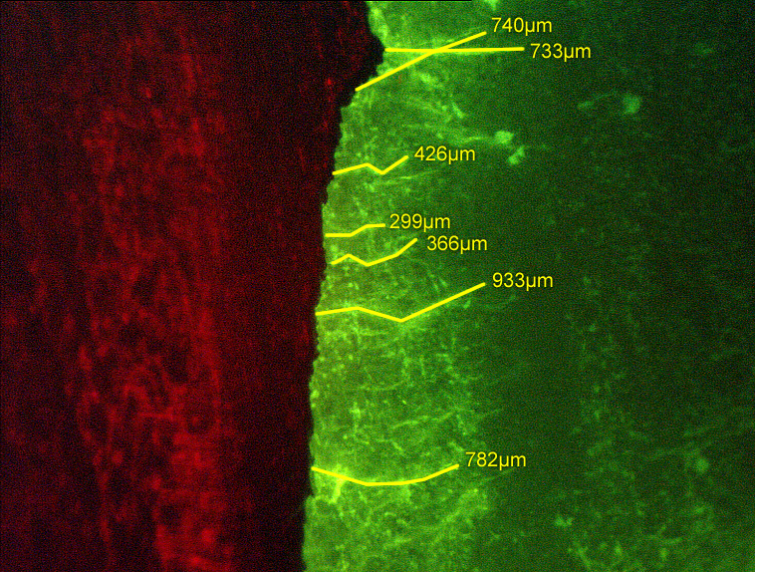
Cracks at the metal (left hand)-cement (right hand) interface made visible using the fluorescence penetration technique.

As with all cyclically loaded systems improving retention stability and reducing debonding tendency means reducing crack, void and flaw frequencies in the interfaces of TKA/bone cement/bone [11]. Cracks originating at the interface metal/bone cement may also influence stability at the interface bone cement/bone, since due to subcritical crack growth promoted by static and in particular due to cyclic loading cracks starting at the interface metal/bone cement can extent to the interface bone cement/bone (distance to be bridged shorter than 2 mm [10]: Fig. 3 reveals one crack which has already extended to 1 mm). Bone cement has brittle properties and crack branching can play an important part. This is similar to other brittle materials like ceramics and glasses [12,13].

"Craquelées" in PMMA full dentures are a well known phenomenon in dentistry. Therefore, both interfaces behave not isolated and independent, they interact instead. Reducing crack frequency at the interface metal/bone cement must have positive influence on the interface bone cement/bone.

Since in vivo conditions of the human body (37°C, 100% humidity) strongly favor hydrolysis, and due to the strong interaction of the metal oxide layer of the metal surface of the TKA with polar water molecules, the bone cement/metal interface is prone to hydrolysis [16]. The water causes hydrolytic degradation of the metal/bone cement interface, when it is not conditioned to this environment [15-20]. The water molecules diffuse through the permeable PMMA and easily reach the interface to form a moisture film at the metal-cement interface even when the PMMA layer and the interface is free of cracks [14]. Any cracks or fissures will accelerate the process via capillary action. Thus, the initially solid bond between the oxide layer of the metal surface and the bone cement is eroded and becomes mechanically unstable as the metal-cement interface is progressively hydrolyzed. The well known radio lucent lines discussed frequently in literature are signs of that phenomenon [21,22].

This hydrolytic debonding, which is largely independent of design of the prosthesis, greatly favors microscopic movements in the metal-cement interface even during normal walking due to repeated pressure and corresponding shear stresses. Step by step interface debonding accompanied by increasing appearance of radio lucent lines results in a vicious circle of cement degradation with cement wear and foreign body-induced osteolysis with eventually mechanical (aseptic) loosening of the TKA.

Hence loosening rates of surface cemented tibial components can be reduced if attention is focused on the central and most common failure mechanism: crack formation and its avoidance [11-13].

This study was done to evaluate a new coating process of cemented tibial components which appears to be suitable to reduce the frequency of crack formation drastically. A biomechanical model for vibration fatigue trials was designed to simulate the physiological and biomechanical conditions of the human knee joint to make the cracks visible.

## Methods

The new coating method [14,24] consists in silicoating the metal surface by sublimating a stream of silicon monoxide particles onto the metal surface under a high vacuum (p = 10^-5 ^mbar and better; PVD method). The particle source was heated to 1130°C. Nevertheless "pre"heating of the tibial component is not to be feared because a distance of about 100 mm between source and substrate was kept and the shutter window between source and substrate was constricted to about 10 mm. The SiO_x _layer is held in place by adhesion (Van der Waals bonds, hydrogen bonds, ionic bonds) and by mechanical microbonds. A silane layer (3-methacryloxypropyltrimethoxysilan) is then applied to the silicoated metal surface [15], and both layers are finally coated with a polymer protective varnish consisting of MMA (methylmethacrylate), UDMA (urethanedimethacrylate) and TEGDMA (triethylene glycol dimethylacrylate) to ensure covalent bonding between the coating and the bone cement. The MMA/UDMA/TEGDMA varnish is then hardened by UV light in a desiccator under vacuum. The coating is stable at normal environmental conditions even it is finally gamma-sterilized at up to 25 kGy. That layer has protective function because it protects the highly reactive silicate/silane coating from all chemical and physical contamination [14]. When applying bone cement to the varnish it is integrated into the bone cement by chemical reaction.

**Figure 2:**
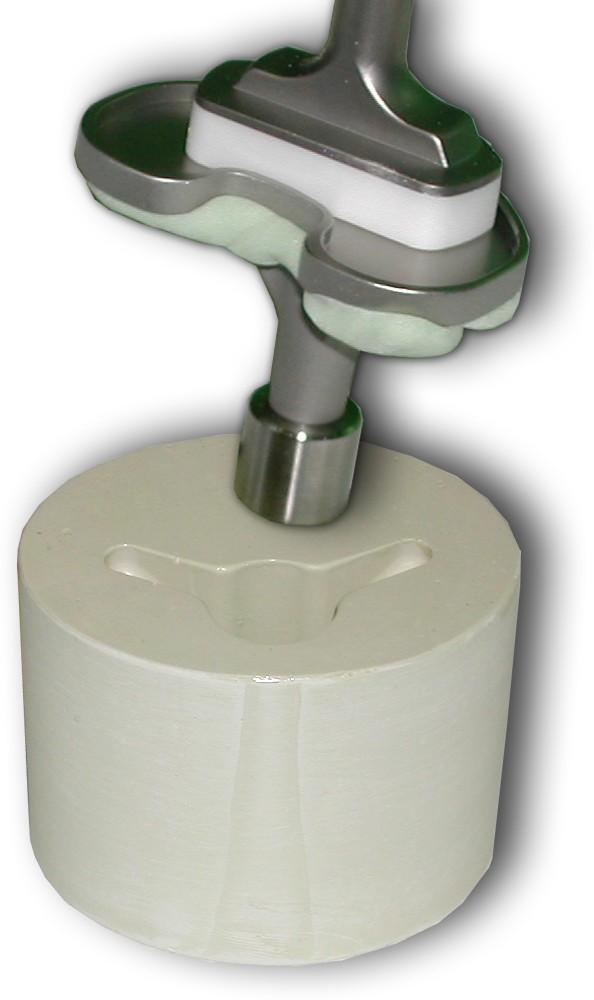
Tibial components ready for surface cementation onto the Technobones (below). Note that the central hole was machined such that there was no "press fit" between the TKA and the Technobones. This ensured maximum stress in the interface.

In order to access the clinical suitability of this patented (European and US patents [24]) coating process for TKA [25], size T3 Columbus tibial components (CoCrMo alloy, for geometry of stem refer to Fig. 2; Aesculap, Tuttlingen, Germany) were tentatively cemented in composite sawbones for biomechanic studies (Sawbones Europe, Malmö, Sweden; note that we did not use "foam bones"; *surface cementing technique applied*). Cementation to cadaver bone was considered not to be feasible for hygienic reasons since we did not succeed in finding a partner which was ready to carry out the saw cuts to be described below. The prosthesis was subjected to a cyclical stress test under near-physiological conditions (37°C, 0.9% NaCl solution) in accordance with ISO 14243. Cyclic loading is considered to resemble the physiological load during walking and it promotes the inception of cracks at voids and flaws and their growth with time in brittle materials [12,13].

This pilot study showed that the sawbones did not withstand a stress of 6 kN. When designing the experiment this load was considered to be adequate since its level did not only reflect the physiological conditions but also supplied an overload to test whether the coating could withstand even larger loads. Retrospective, a load of 3 kN would have been a better choice (corresponds to a person of 75 kg of weight [26]). Results for sawbones are closer to the physiological situation.

We switched over to very stable "technobones" which could withstand the stress of 6 kN. The "technobones" consisted of Technovit^® ^4002 (Heraeus-Kulzer, Werheim, Germany). Since its main component besides styrol and dimethylanilin is MMA good bonding to bone cement is assured due to near chemical neighborhood. Technovit "powder" and Technovit "liquid" were mixed at a weight ratio of 2:1 and poured into a mold to achieve similar contours as known from the sawbones. In order to improve their wettability by the bone cement when mounting the tibial components the proximal surface of the "technobones" was coated with the varnish described above.

Young's moduli of the sawbones were E = 15 GPa and E = 0.5 GPa for the cortical surface and the spongeous inner part, respectively. In order to check whether young's modulus of the "technobones" was similar to that of the sawbones we prepared five rods (length: 55 mm, width: 6 mm, thickness: 3 mm) which were subjected to a tensile stress test (universal testing machine Zwick 030, Zwick, Ulm, Germany). Using the standard formula Young's modulus was found to be E = 1.4 ± 0.6 GPa, similar to the spongeous inner parts of the sawbones.

**Figure 1:**
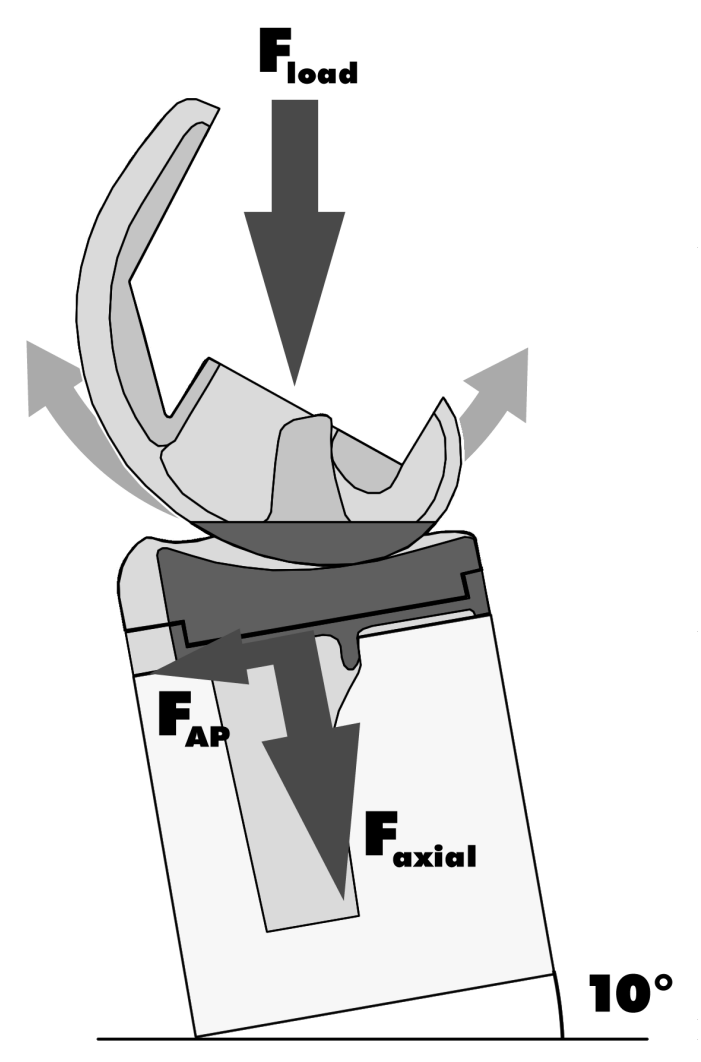
Load geometry for servopneumatically controlled 6kN two-column tabletop tester.

The vibration fatigue test was considered to be a suitable biomechanical model (geometry of load: refer to Fig. 1). It was done using a servo pneumatically controlled 6kN two-column tabletop tester (Dyna-Mess, Aachen, Germany). The samples (tibial components cemented to the "technobones": Fig. 2; surface cementing technique applied, no press-fit, stem neither conditioned nor cemented) were kept in a plexiglass chamber under physiological conditions, i.e., at 37°C and at 100% relative humidity while being immersed in a 0.9% NaCl solution. This environmental conditions ensured hydrolytic conditions similar to the human body.

The test arrangement was mounted on ball bearings for free movement in the anterior-posterior direction and to eliminate all transverse forces while allowing rotation. Inclination of the tibial plane was 10°.

Each tibial implant was exposed to 10^6 ^alternating (sine) stress cycles, with a minimum and maximum load of 150 N and 6,000 N, respectively and a cycle frequency of 3 Hz to simulate the physiological conditions in the knee over an average walking period of one year. Total test duration for one component was 3.86 days (about 93 hours). A total number of 8 coated (before PVD-layering abraded by Al_2_O_3_-blasting (grain size 120 μm) and 12 uncoated (F22 (R_a _= 3.41 μm) = 8, Silbermatt (R_a _= 0.49 μm) = 2, B60 (R_a _= 1,96 μm) = 2; P1 and P2) tibial components were tested. After the vibration fatigue trials, the components were removed and kept in a wet atmosphere at room temperature, as drying was shown reducing crack frequencies and restoring bonding by virtue of water leaving the interface.

For evaluation, the prostheses were sliced vertically using a high-pressure water jet (IPT, Frauenhofer-Insitut, Aachen, Germany), cleaned from debris and air-dried. Afterwards the surface was microscopically examined for cracks at the metal-cement interface using the fluorescent penetration technique (Fig 3). A penetration fluid (MET-L-CHEK Penetrant FBP 913, HELLING, Heidgraben, Germany) with a high capillarity and a low surface tension enters cracks in the surface. Scanning the area with a UV light microscope (Leica DMR X, Leica, Wetzlar, Germany; approx. wavelength 360 nm) revealed cracks even as narrow as 0.25 μm. To detect gaps the surface was investigated using standard microscopic technique (reflected-light microscopy).

## Results

**Figure 4:**
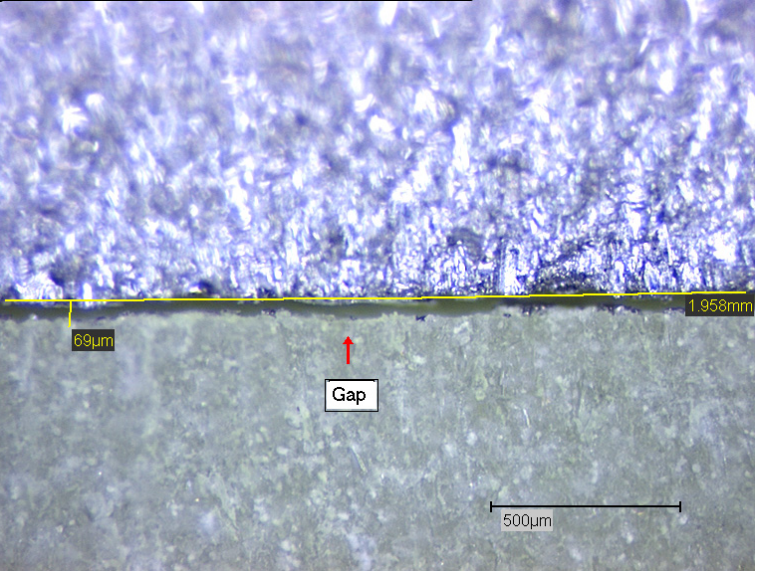
Gap at the metal (above)-cement (below) interface.

The length and maximum width of all cracks and gaps were recorded. Fig. 3 shows an example of a crack area and Fig. 4 an example of a gap. However, the overall number of gaps detected was much smaller than the number of cracks. Hence we focused to the number and quality of cracks utilizing their numbers and quality as criterions to differentiate between coated and uncoated samples.

The coated components showed almost no cracks. The cumulative crack length of them was 937 μm while the uncoated components showed cumulative crack lengths of 13,999 – 27,351 μm (Table 1).

**Table 1:** Cumulative crack lengths for various prostheses

**Prosthesis**	**Length [μm]**
Silbermatt (m = 2)	13,999
F22 (m = 8)	24,693
B60 (m = 2)	27,351
Coated (m = 8)	937

The differences in cumulative crack lengths between the firstly tested "P1-B60" components (13,180 μm) and the secondly tested "P2-B60" (41,521 μm) components are due to the fact that both the P1-B60 and P2-B60 interfaces detached from the metal surface during the trial (the P1-B60 interface in an early stage and the P2-B60 interface towards the end). There were clearly more lateral than medial cracks.

The coated components showed the best overall performance (Table 2, 0.8%). There were no significant differences between uncoated, Silbermatt (R_a _= 0.49 μm, 1.7%) and uncoated F22 (R_a _= 3.41 μm, 2.5%) components, although the latter both as a group differed greatly from the B60 (R_a _= 1.96 μm, 69%) components. The B60 (m = 2) components also showed a significant difference between medial (49.3%) and lateral (87.7%) cracks, which the other types did not (Silbermatt (m = 2): 1.7% vs. 1.7%; F22 (m = 8): 2.2% vs. 2.7%; coated (m = 8): 0.9% vs. 0.8%).

**Table 2:** Relative proportion of gap formation by prosthesis type

**Coated Prostheses**	all	0.8 %
**Uncoated Prostheses**	Silbermatt (m = 2)	1.7 %
	F22 (m = 8)	2.5 %
	B60 (m = 2)	69.0 %

**Figure 5:**
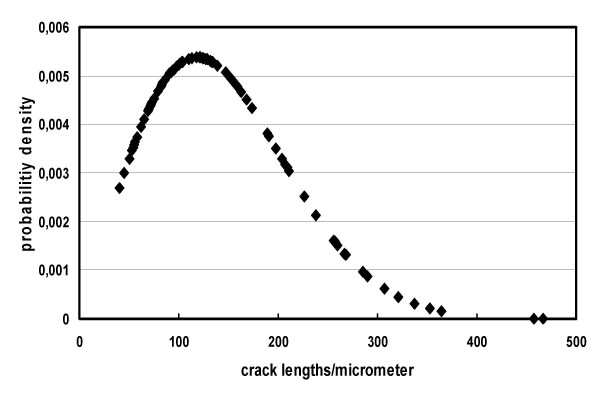
Prosthesis with surface specification B60: Weibull distribution of crack lengths. Modal value π = 120 μm (crack with maximum propability P). Median value of crack length δ = 163 μm (P = 63%; from Fig. 6). Note that the values given in Tab. 3 for B60 are slightly different (170 μm and 133 μm for Median and Modal value, respectively) since those values represent the arithmetic means of characteristic values determined for all prosthesis of this type.

**Figure 6:**
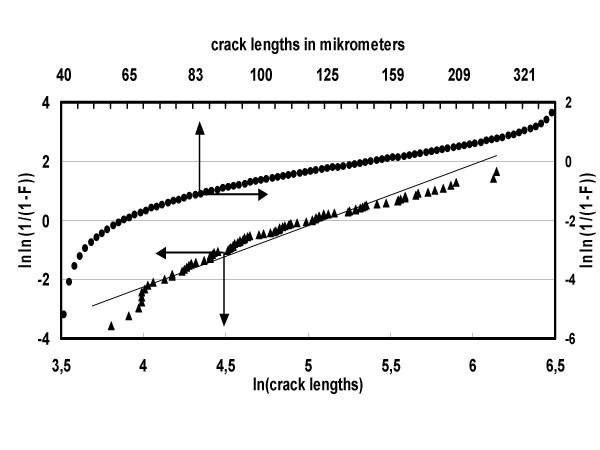
Prosthesis with surface specification B60: Weibull plot of crack length. Median value of crack length μ = 163 μm. Modal value of crack length π = 120 μm (from Fig. 5). Modul m = 2,1.

Since there were more short than long cracks, distribution was asymmetrical. This suggested a Weibull statistical analysis (examples and graphical method: Fig. 5 and Fig. 6; summary of results: Table 3) which yielded for Silbermatt components a median of 260 μm, a modal value of 175 μm and the lowest number of cracks (52). The F22 had a median of 239 μm, a modal value of 205 μm and 112 cracks. The B60 components, the interfaces of which were partially debonded on a macroscopic scale during the vibration fatigue trial, had a median of 170 μm, a modal value of 133 μm and 177 cracks (refer also to Fig. 5 and Fig. 6). All the components types have almost the same Weibull shape parameter m = 2. "Median" means that half of the crack lengths will be less than this value and half will be larger. The modal-value gives the crack length with the highest probability and the shape parameter, m, tells how blurred the distribution is, i.e. if all the crack lengths tend to be not very close to a certain value, the distribution will have a low shape parameter m, and the distribution appears to be very flat and blurred.

**Table 3:** Characteristic Weibull parameters of uncoated prostheses

**Prostheses**	**Area**	**Median δ [μm]**	**Modal π [μm]**	**Number n**
P_Silbermatt_	all	260	175	52
P_F22_	all	239	205	112
P_B60_	all	170	133	177

## Discussion

Our findings show that mechanical degradation in combination with hydrolytic load of the interface metallic tibial surface/bone cement is an important failure mechanism for aseptic loosening of surface cemented tibial prosthesis components. From previous investigations we have learnt [14] that the bond strength between bone cement and unconditioned samples of CoCrMo is unstable against hydrolysis [15,16] and stability is limited by retention due to surface roughness. PVD conditioned samples (silicoating, silane, coating varnish), however, reveal stable bond strengths even in a humid surrounding [23]. These results collected from investigations on a sample geometry which is most suitable to measure bond strengths under tension load (adhesion means resistance against normal loads; these are aligned perpendicular to the adhesion surface) appear to become resembled by our investigations on real tibial components. Without PVD layering and varnish implant stability is limited by the retentive micro- and macro mechanical anchoring of the PMMA polymer to the corundum blasted metal surface. Since no chemical bonding has taken place, the metal surface is prone to hydrolytic degradation [14]. In addition cracks and gaps arise in the bone cement, starting from irregularities in a surface near PMMA layer and from voids in the balk of the PMMA [11]. Irregularities in a surface near the PMMA (e.g.) layer are mainly due to the retention mechanism which is typical for a material combination of a rough metal and a plastic layer. The corundum grains at high speed blasted onto the surface of the metal score deeply into the surface, creating furrows which are bordered by rough, upturned edges. The burrs cut into the plastic and if loaded they provide weak links (predetermined breaking points). The weak links result in cracks and extend due to cyclic load in analogy to subcritical crack propagation in ceramics leading to further deterioration of the cement layer, PMMA abrasion and (in vivo) to foreign body-induced resorption at the bone matrix, increasing the micro movements in a vicious circle, eventually making replacement necessary.

In the contrary, when the rough surface is PVD layered by a SiO_x _film the surface becomes much smoother and the burrs and edges become masked by this layer. Moreover, there is no longer the need of a rough surface with deep cuts. A textured surface for an increase of the effective surface is sufficient. The weak links disappear then or its number is reduced. The surface is no longer prone to emit cracks into the PMMA cement in abundance.

Hence, in order to reduce or even prevent the described mechanisms of degradation and loosening, the prosthesis surface of knee implants must be modified to provide a more durable, hydrolysis-proof interface between bone cement and metal. Strong covalent bonds between bone cement and the prosthesis prevent micro movements at the interface, and hence formation of cracks and aseptic loosening of the implant.

Similar problems are encountered in the field of prosthodontics with veneering crowns and bridges by plastics, where the solution was a firmly adhering layer of silicate [23]. After applying this principle to cemented hip endoprosthesis, the present investigation showed that it became possible to create a durable, hydrolysis-resistant interface between the metallic prosthesis and the bone cement polymer, thus probably improving the life of endoprosthesis at a rate not yet known [25].

The vibration fatigue trials at 10^6 ^stress cycles and after 7 days in physiological (0,9 %) NaCl solution, done to simulate downhill walking, showed that under standardized and reproducible in-vitro conditions, the coating system withstands the typical mechanical stress present under physiological conditions. The results were confirmed by interface analysis. No cracks were found in three of the eight coated components, the other showed only minimal cumulative crack lengths (937 μm), whereas the uncoated components F22 showed significantly more and longer cracks (Table 1). Comparing the surface roughness of the uncoated components, it was seen that the Silbermatt components exhibited lower cumulative crack lengths than the F22 components while the B60 components showed a fatal crack spectrum and partial macroscopically debonding of the metal-bone interface.

Analyzing the crack locations along the medial and lateral lines of symmetry clearly revealed more lateral than medial cracks. Parting from the mediolateral distribution of load (67.5% medially and 32.5% laterally), it becomes apparent that the cracks are caused more by tensile than by compressive stress. These results agree with reports in the literature. Bone cement has a higher compressive than tensile strength (80 MPa vs. 30 MPa), the latter thus being critical in causing material failure [25].

The coated components showed the best overall performance, with a gap formation ratio of 0.8%, whereas the uncoated components showed more variable rates (Table 2). Due to the partial debonding and interface deterioration of the B60 components, the crack rate was 69%. The results clearly show that the new coating system is well suited for preventing hydrolytic debonding at the metal-bone interface.

Since there were more smaller than longer cracks, an asymmetrical crack spectrum originated. This suggested to endeavor a Weibull analysis of crack distribution. This kind of analysis revealed to be very suitable for this purpose because it enabled us to list median and modal values characterizing the crack spectra of the uncoated components investigated.

By the coated components we could not employ the Weibull distribution because they revealed almost no cracks. By the other component types one sees that the Silbermatt ones have done best. They have the smaller modal value and the lowest number of cracks in comparison to the F22 one (Table 3).

Earlier efforts to decrease the rate of aseptic loosening, however for femoral components, resulted in a considerably higher rate of failure with failure occurring earlier. The components were designed to have a more roughened or textured surface and a PMMA precoating [27,28]. Note that under chemical aspects there is no reason why a PMMA precoating should have a higher resistance against hydrolysis than a surface near PMMA layer which is part of the cementing. To make a surface more rough is of no use because the above described effect of burrs will be more pronounced and the failure due to crack development and crack propagation will occur earlier.

## Conclusion

There is a dilemma to be solved: Cementing the stem means improved retention and additional orientation for the tibial component. However stress shielding occurs which is an unfavorable condition for a healthy bone. In case of revision additional bone is lost.

Only cementing the surface of the tibial component means largely reduced overall retention area and loss of orientation for the tibial component. This is reflected by the loosening rate of 10.5 % after an average interval of four years [1]. The rate may be diminished by "press fitting" the stem.

In the present contribution we have demonstrated how this dilemma can be solved: gaining additional retention for the tibial surface by application of a layer system which stabilizes the retention under hydrolytic and cyclic load and which helps avoiding the initiation of cracks. Then cementing of the stem can be abandoned and a more physiological load of the tibia head is reached. Stabilizing the interface metal/bone cement also means indirectly stabilizing the interface bone cement/bone since the frequency of cracks which emanate from the metal surface and propagate through the bone cement to the bone surface is dramatically reduced by the proposed and patented layer system.

Is should be kept in mind that for the presently discussed results instead of natural bones we utilized artificial "techno"bones. Therefore, a multicentre study with several university clinics is going ahead to validate these results in vivo.

A particular interesting aspect will be to notice whether also the frequency of radio lucency is be reduced for coated tibial components.

## Authors' contributions

RM conceived in the study, participated in the design, carried out the statistical analyses and drafted the manuscript. MQ carried out experimental work. DCW conceived in the study and participated in the design. FUN coordinated the work. TM carried out experimental and analytical work and participated in drafting the manuscript. All authors read and approved the final manuscript.
